# Validation of a Single-Nucleotide Polymorphism-Based Non-Invasive Prenatal Test in Twin Gestations: Determination of Zygosity, Individual Fetal Sex, and Fetal Aneuploidy

**DOI:** 10.3390/jcm8070937

**Published:** 2019-06-28

**Authors:** Errol R. Norwitz, Gabriel McNeill, Akshita Kalyan, Elizabeth Rivers, Ebad Ahmed, Ling Meng, Phikhanh Vu, Melissa Egbert, Marlene Shapira, Katie Kobara, Sheetal Parmar, Shruti Goel, Sarah A. Prins, Israel Aruh, Nicola Persico, Jared C. Robins, Brian Kirshon, Zachary P. Demko, Allison Ryan, Paul R. Billings, Matthew Rabinowitz, Peter Benn, Kimberly A. Martin, Herman L. Hedriana

**Affiliations:** 1 Tufts Medical Center and Tufts University School of Medicine, Boston, MA 02111, USA; 2 Natera, Inc., San Carlos, CA 94070, USA; 3 Dr. Israel Aruh’s IVF and Infertility Clinic, 35220 Izmir, Turkey; 4 Ospedale Maggiore Policlinico, 20122 Milan, Italy; 5 Northwestern University, Feinberg School of Medicine, Chicago, IL 60611, USA; 6 Houston Perinatal Associates, Houston, TX 77054, USA; 7 UConn Health, Farmington, CT 06030, USA; 8 University of California Davis Health, Sacramento, CA 95819, USA

**Keywords:** non-invasive prenatal testing, twins, zygosity, aneuploidy, Down syndrome, prenatal screening, chorionicity

## Abstract

We analyzed maternal plasma cell-free DNA samples from twin pregnancies in a prospective blinded study to validate a single-nucleotide polymorphism (SNP)-based non-invasive prenatal test (NIPT) for zygosity, fetal sex, and aneuploidy. Zygosity was evaluated by looking for either one or two fetal genome complements, fetal sex was evaluated by evaluating Y-chromosome loci, and aneuploidy was assessed through SNP ratios. Zygosity was correctly predicted in 100% of cases (93/93; 95% confidence interval (CI) 96.1%–100%). Individual fetal sex for both twins was also called with 100% accuracy (102/102; 95% weighted CI 95.2%–100%). All cases with copy number truth were also correctly identified. The dizygotic aneuploidy sensitivity was 100% (10/10; 95% CI 69.2%–100%), and overall specificity was 100% (96/96; 95% weighted CI, 94.8%–100%). The mean fetal fraction (FF) of monozygotic twins (*n* = 43) was 13.0% (standard deviation (SD), 4.5%); for dizygotic twins (*n* = 79), the mean lower FF was 6.5% (SD, 3.1%) and the mean higher FF was 8.1% (SD, 3.5%). We conclude SNP-based NIPT for zygosity is of value when chorionicity is uncertain or anomalies are identified. Zygosity, fetal sex, and aneuploidy are complementary evaluations that can be carried out on the same specimen as early as 9 weeks’ gestation.

## 1. Introduction

Twin gestations account for approximately 1 in 30 live births in the United States [1] and have a 4- to 10-fold increased risk of perinatal complications compared to singleton pregnancies [2]. Monochorionic (MC) twins account for ~20% of twin gestations [3,4] and have higher rates of major structural defects, miscarriage, preterm delivery, and selective fetal growth restriction compared to dichorionic (DC) twins [5]. Of particular importance, MC twins are at high risk for twin-to-twin transfusion syndrome (TTTS), which accounts for more than one-third of perinatal MC twin deaths [6,7]. 

Early establishment of zygosity can prompt increased surveillance of cases at high risk for TTTS and other MC twin-related abnormalities [8]. Ultrasound examination performed in the first trimester or early second trimester can distinguish between MC and DC twins, but this is less reliable later in pregnancy [9]. Because dizygotic (DZ) (non-identical) twins almost always have a DC placenta, establishing zygosity through genetic testing could potentially aid in patient management. An accurate non-invasive prenatal test (NIPT) for zygosity would be particularly advantageous for women presenting later in pregnancy, when there is a fetal abnormality or aneuploidy marker in one of a same-sex pair, or where there is MC/DC uncertainty based on an early ultrasound examination. Accurate establishment of zygosity can be achieved through analysis of single-nucleotide polymorphisms (SNPs) from whole blood, dried blood spots, and saliva [10] and an initial proof-of-principle report demonstrated the feasibility of prenatal cfDNA-based zygosity determination (*n* = 8) [11]. 

Zygosity testing and the evaluation of risk for fetal aneuploidy can be conducted on the same sample. Aneuploidy screening is of particular value in DZ pregnancies because the per pregnancy risk (i.e., the probability that at least one fetus will be affected) is increased relative to the risk in singleton pregnancies [12,13]. Moreover, DZ twinning is more common in older women and these women will therefore have higher prior risk [1]. 

Non-invasive prenatal testing (NIPT) has been developed as a screening test for fetal aneuploidy in twin pregnancies. Meta-analyses of NIPT in twin pregnancies have shown high sensitivity and specificity for trisomy 21 [14,15], with test performance superior to traditional screening methodologies [16]. 

In this study, we validate the performance of a single-nucleotide polymorphism (SNP)-based NIPT to assign zygosity, evaluate the accuracy of sex determination, and present preliminary data on fetal aneuploidy screening in twin gestations.

## 2. Materials and Methods

Pregnant women (≥18 years of age) with sonographically confirmed twin pregnancies were enrolled at 21 locations in compliance with local laws and institutional review board-approved protocols (Supplementary Methods 1) (Western IRB Protocol number 20130376). Following informed consent, 20 mL of maternal blood was collected; samples were analyzed at a Clinical Laboratory Improvement Act (CLIA)-certified and College of American Pathologists (CAP)-accredited laboratory (Natera, Inc.; San Carlos, CA, USA) using an SNP-based NIPT methodology described previously [17,18,19]. Samples were accumulated from April 2013 to February 2017 and held frozen prior to this prospective trial. All cases and the corresponding independently determined confirmatory data for zygosity, fetal sex, and aneuploidy status of the pregnancies (Supplementary Methods 2) were de-identified prior to NIPT analysis such that the testing was carried out in a blinded manner. 

Zygosity, aneuploidy status, and fetal sex NIPT calls were made using a version of a previously validated algorithm for singleton pregnancies [17,18,19,20], with modifications for twin gestations that were prespecified prior to initiation of the current validation study. All cases meeting the test eligibility criteria were included in the study. Not all cases had confirmation data for all three test components (zygosity, fetal sex, and aneuploidy); only cases with confirmatory data for a given component were evaluated for that indication. Thus, there were three overlapping cohorts consisting of 95 (30 monozygotic (MZ), 65 DZ) cases for zygosity determination, 103 (40 male/male, 35 male/female, 28 female/female) cases for fetal sex determination, and 117 cases (11 aneuploid and 106 euploid cases) for aneuploidy testing. The total number of twin pregnancies in the study was 126 (Supplementary Table S1 and Supplementary Figure S1). Patient demographics and characteristics are detailed in Supplementary Table S2. Samples with multiple aneuploidy conditions within a twin pregnancy were excluded. 

The sensitivity, specificity, and accuracy of the test in detecting zygosity, aneuploidy, and individual fetal sex were determined on samples that received a call. First, zygosity testing was performed; subsequently, analysis for fetal sex and aneuploidy was carried out with prior risk adjusted based on zygosity status (based on clinical truth or, if it was not available, the output of the SNP-based algorithm). Overall performance metrics were estimated using a weighted average based on the population prevalence of MZ:DZ twin pregnancies (30:70) [21]. Study analysis was conducted at the laboratory using a commercial (non-research) software environment. Calls were generated by the algorithm and data were reviewed when necessary according to a predetermined standard operating procedure. 

**Figure 1:**
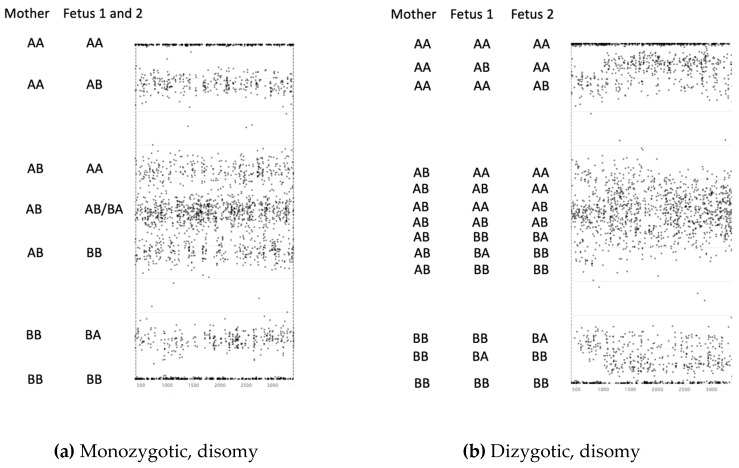

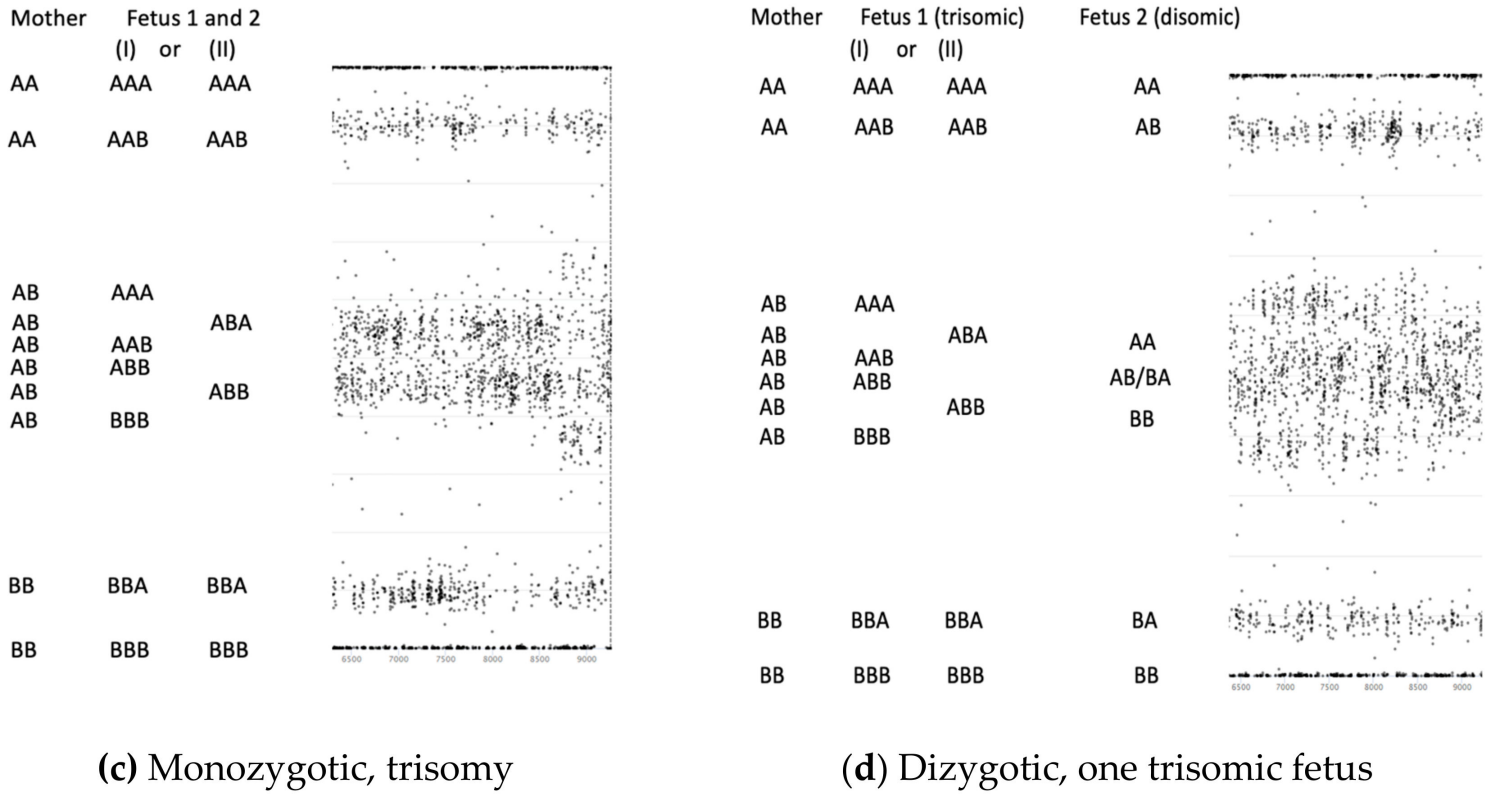
Heterozygosity plots for SNP alleles for a single chromosome in twins. Legend: *x*-axis—relative position of the SNP along the chromosome; *y*-axis—relative amount of the alleles (100% A at the top and 100% B allele at the bottom). (**a**) The pattern of SNPs in monozygotic twins is the same as in singleton pregnancies as there are only two different genotypes present. The relative vertical position of heterozygous alleles is determined by the fetal fraction, with two SNP bands at the top, three in the middle, and two at the bottom. (**b**) Dizygotic twins share zero, one, or two homologous regions of their genomes, depending on the recombination and inheritance patterns. The pattern for dizygotic twins differs from monozygotic twins over those regions where the twins share zero or one homologous regions due to the extra genotype present in the DNA mixture. Over those regions, there are more possible combinations alleles at a given SNP, meaning more bands. In this example, the fetal fraction for fetus 1 is less than that for fetus 2. (**c**) When trisomy is present in a monozygotic twin pregnancy, the number of bands change, with the number depending on the number of homologous regions from each parent. The pattern is similar to a singleton pregnancy where trisomy is present. Two alternative fetal genotypes are shown; I depict the situation where two homologs are inherited from the father, or where two identical homologs are inherited from the mother, and II is where one of each of the maternal homologs are present in the fetus. The pattern can change along the chromosome as a result of recombination. (**d**) The combination of a trisomy and disomy in a dizygotic twin pregnancy results in a complex pattern. Although more complex, the informatics can compute the two FFs and determine the presence of trisomy through comparison of relative SNP amounts across chromosomes.

Zygosity calls were made by evaluating up to 12,568 SNPs with a pattern compatible with a single set of fetal alleles indicative of an MZ pregnancy or, alternatively, a pattern compatible with two sets of fetal alleles for a DZ pregnancy. Figure 1 illustrates how the allele distributions differ between monozygotic and dizygotic twins and explains how aneuploidy and fetal fraction can be determined from these data. For each sample, the SNPs are evaluated against the two hypotheses: first, that they are from an MZ, and second, a DZ pregnancy. For an MZ pregnancy, the interpretation of the SNP pattern is essentially the same as for a singleton pregnancy. For a DZ pregnancy, the non-maternal alleles are interpreted as being derived from one or both fetuses. A paternal sample was not required for the test interpretation. MZ sensitivity was estimated as the proportion of confirmed MZ pregnancies with a valid zygosity call that were called correctly. DZ sensitivity was estimated as the proportion of confirmed DZ pregnancies with a valid zygosity call that were called correctly. Zygosity test accuracy was estimated as the proportion of pregnancies with a valid zygosity call that were called correctly.

Fetal fraction (FF) estimates were determined for both fetuses based on the allele ratios: A combined FF for MZ twins using the same methodology as for singletons and two distinct FF estimates for DZ twins. An FF <2.8% for a putative DZ fetus or a combined FF <2.8% for a putative MZ pregnancy was too low for the test to provide a result. 

Fetal sex assessment was based on the analysis of 277 Y-chromosome loci. The number of male and female fetuses in each twin pregnancy was determined by relating the proportion of Y chromosomal DNA to the observed FF. Two males were called when the amount of Y chromosomal DNA was consistent with the sum of FFs; one male and one female were called when the amount of Y chromosomal DNA was consistent with one of the FFs; two females were called when no Y chromosomal DNA was detected. MZ fetal sex accuracy was defined as the proportion of MZ samples with a valid call that were correctly called as “two males” or “two females” for which confirmed gender information on both fetuses was available. DZ fetal sex accuracy was defined as the proportion of DZ pregnancies with gender truth on both fetuses that received a valid call and were correctly called as either “two males,” “one male and one female,” or “two females.” The overall fetal sex accuracy was computed using the weighted average of MZ and DZ cases that received a call. Fetal sex was only analyzed for cases with an aneuploidy result.

Aneuploidy assessment in MZ twins followed the same methodology used for singleton pregnancies [22]. DZ twins were only assessed for trisomies 13, 18, and 21. A priori risk for an aneuploid pregnancy was based on maternal age-specific rates for singleton pregnancies, with adjustments based on the assigned zygosity (Supplementary Methods 3) [12]. A risk of >1/100 for trisomy 21, trisomy 18, or trisomy 13 was considered a high-risk result. A pregnancy was considered “screen positive” if a copy number abnormality was suspected in at least one fetus and “screen negative” when no abnormality was suspected in either fetus; the methodology did not assign a fetus-specific aneuploidy risk. Aneuploidy sensitivity and specificity (MZ and DZ) and overall aneuploidy specificity using an MZ:DZ weighted average were estimated. In addition, MZ specificity of the test in detecting monosomy X was estimated using a >1/100 risk as a cut-off (Supplementary Methods 4). Generally, zygosity testing could be carried out at lower FF than is possible for aneuploidy detection. Therefore, chromosomal abnormalities were only analyzed for cases when zygosity calls were available. 

Samples that did not return a result for a given test were excluded from the corresponding sensitivity, specificity, or accuracy calculations; repeat samples were not requested (Supplementary Methods 5). Confidence intervals (CI) were computed for all test performance estimates (Supplementary Methods 6).

## 3. Results

### 3.1. Determination of Twin Zygosity

**Figure 2:**
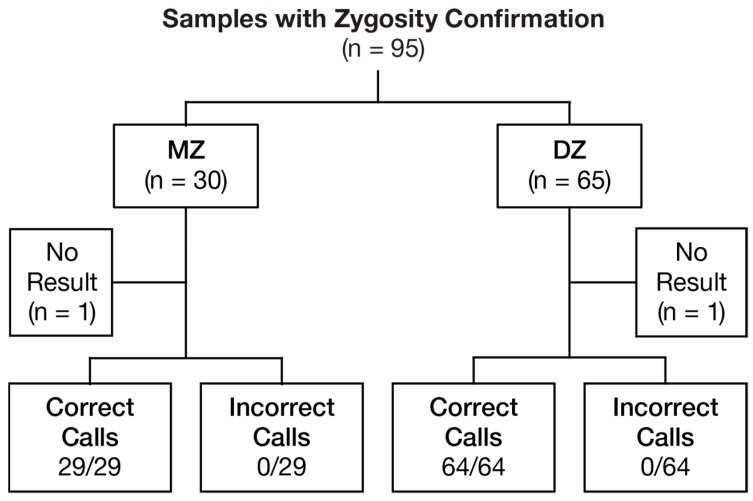
Zygosity determination. MZ—monozygotic; DZ—dizygotic.

To validate the performance of the test for zygosity detection in twin gestations, 95 samples with confirmed zygosity were evaluated (30 MZ and 65 DZ) (Figure 2). All samples that received results (MZ: 29/30 (96.7%) and DZ: 64/65 (98.5%)) were called correctly, yielding an observed MZ sensitivity (DZ specificity) of 100% (29/29; 95% CI, 88.1%–100%) and an MZ specificity (DZ sensitivity) of 100% (64/64; 95% CI, 94.4%–100%). The overall zygosity test correct call proportion was 100% (93/93; 95% CI, 96.1%–100%). Two samples (MZ: 1/30 (3.3%) and DZ: 1/65 (1.5%)) did not receive results due to low FF, generating an overall estimated no-result rate of 2.1% (0.3 × 1/30 + 0.7 × 1/65; 95% weighted CI, 0.65%–8.4%).

### 3.2. Fetal Fraction

**Figure 3:**
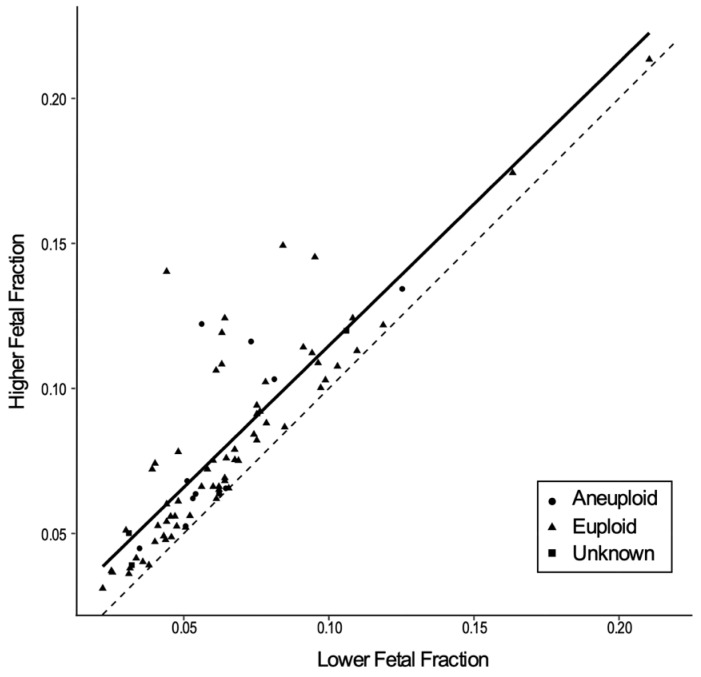
Scatterplot of fetal fraction estimates of dizygotic twins. Legend: Scatterplot of higher and lower fetal fraction estimates of dizygous twin samples (*n* = 79). Samples include cases that were aneuploid (circles) and euploid (triangles). Presence or absence of chromosome abnormality was unknown for three cases (squares). Aneuploid samples include cases affected with either T21, T18, or T13. The solid line represents the linear regression line; the dotted line represents the line of equal fetal fractions (reference 45-degree line). The Pearson Correlation Coefficient was 0.860 (95% CI, 0.736–0.937).

The mean FF of MZ twins (*n* = 43) was 13.0% (SD, 4.5%); for DZ twins (*n* = 79) the mean lower FF was 6.5% (SD, 3.1%) and the mean higher FF was 8.1% (SD, 3.5%). Figure 3 displays a scatterplot of the high and low FF estimates of DZ samples (*n* = 79). The Pearson correlation coefficient estimate between the FF estimates for each pair of FFs was 0.860 (95% bootstrap CI, 0.736–0.937). Of the DZ cases, 20.3% (16/79) had an FF difference of greater than 2% between the two fetuses and 11.4% (9/79) had an FF difference of greater than 4%. All cases with confirmed zygosity that were included in one or more test groups (zygosity, fetal sex, and aneuploidy) were included in the analysis.

### 3.3. Determination of Fetal Sex

**Figure 4:**
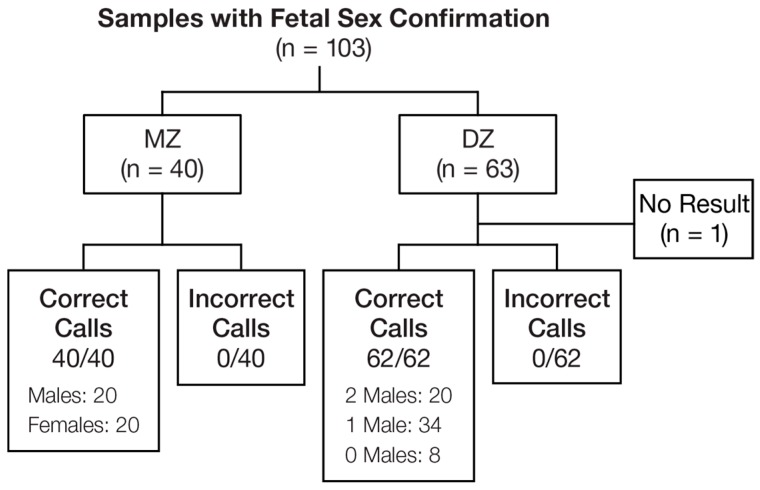
Fetal sex determination. MZ—monozygotic; DZ—dizygotic.

Samples with independently determined fetal sex (*n* = 103) were evaluated to estimate the accuracy of the test for individual fetal sex determination (Figure 4). All cases that received a result were called correctly, including 40 MZ cases (two males (20/20), two females (20/20)) and 62 DZ cases (two males (20/20), one male and one female (34/34), two females (8/8)). The overall accuracy to determine fetal sex was 100% (102/102; 95% weighted CI, 95.2%–100%). One DZ case did not receive a result due to low FF, yielding a no-result rate of 1.1% (0.3 × 0/40 + 0.7 × 1/63; 95% weighted CI, 0.03%–6.6%).

### 3.4. Screening for Chromosome Abnormality

**Figure 5:**
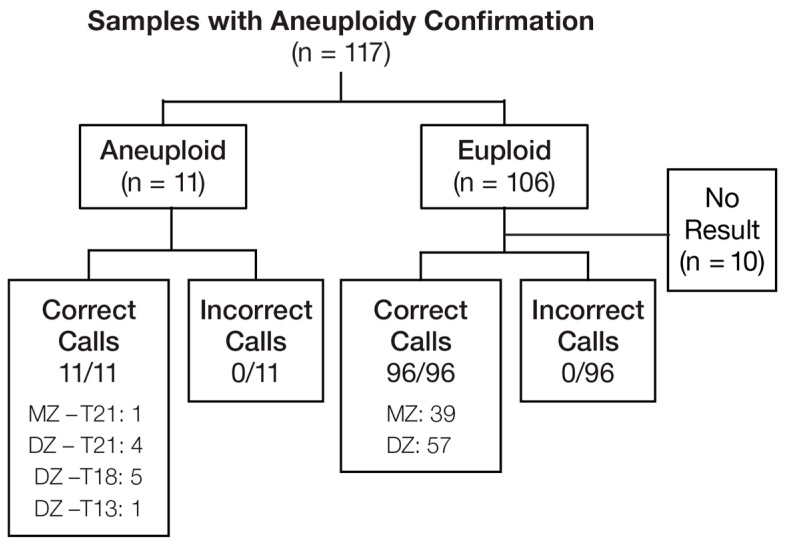
Aneuploidy determination. MZ—monozygotic; DZ—dizygotic.

The evaluation of the performance of the testing for chromosomal abnormalities was based on a total of 117 cases, including 106 euploid samples, 5 T21 (1 MZ and 4 DZ), 5 T18 (all DZ), and 1 T13 (DZ) (Figure 5). All 11 aneuploidy-affected samples were correctly detected. For DZ twin gestations, the sensitivity was 100% (10/10; 95% CI, 69.2%–100%). Ninety-six samples (39 MZ and 57 DZ) that were confirmed euploid received a result; all were accurately called as negative, yielding an overall aneuploidy specificity of 100% (96/96; 95% weighted CI, 94.8%–100%). Of 87 euploid samples with gestational age (GA) ≥10 weeks, 0/21 (0%) MZ samples and 10/66 (15.2%) DZ samples did not receive a result, yielding an estimated overall no-result rate of 10.6% (0.3 × 0/21 + 0.7 × 10/66; 95% weighted CI, 5.3%–19.7%).

## 4. Discussion

In this study, we report the validation of a SNP-based NIPT methodology to assign zygosity, determine individual fetal sex, and detect aneuploidy as early as 9 weeks of gestation. The three complementary pieces of information can be assessed in parallel in twin pregnancies with implications for improving prenatal management.

Accurate determination of zygosity is beneficial, particularly in women with uncertainty concerning chorionicity, which is a key factor that impacts the prognosis of twin gestations. The importance of this information early in pregnancy is well established [23]. MC twin pregnancies share their blood supply and up to 15% of these cases are affected by TTTS [7]. Furthermore, MC pregnancies are also at high risk for twin anemia–polycythemia sequence, fetal anatomic abnormalities, fetal growth restriction, and prematurity. 

Monochorionic/monoamniotic (MCMA) twins, although rare (<1% of MZ twins), are at the highest risk of adverse outcomes, primarily due to additional complications associated with cord entanglement [24]. MC pregnancies require complex prenatal surveillance, at a minimum of every two weeks beginning in the second trimester [25]. Twin pregnancies with dichorionic/diamniotic (DCDA) placentation have the most favorable outcome, although risk for pregnancy complications remains higher than that for singleton pregnancies. 

Chorionicity assignment through ultrasound is the current gold standard during pregnancy and has high specificity and sensitivity in the first trimester. However, the accuracy of ultrasound for chorionicity establishment is both operator- and time-dependent. When performed in the late first/early second trimester, up to 19% of high-risk MC twins were incorrectly classified as DC [26]. DZ twins, which make up 70% of all twin pregnancies, predominantly have DC placentation. In contrast, MZ twins may either have a DC (20–30%) or MC (70–80%) placentation. Establishing zygosity can therefore provide an alternative source of information to assess risk in twin pregnancies and this would be of particular value for women who do not receive first trimester ultrasound. Zygosity determination needs be combined with FF determination for both fetuses in order to avoid interpreting a DZ pregnancy where one fetus has very low FF as an MZ pregnancy. We show here that the SNP-based NIPT can be used for the accurate determination of zygosity as well as determination of individual fetal sex as early as 9 weeks of gestation. To our knowledge, this is the first validated NIPT to determine zygosity.

We also report on the performance of SNP-based NIPT in screening for fetal aneuploidy in twin pregnancies. Determination of robust sensitivity estimating the detection of aneuploidies, such as T13, T18, and monosomy X, is challenging due to the rarity of affected twin pregnancies. Knowledge of the zygosity status for evaluation of aneuploidy risk is advantageous because the prior risk of an affected pregnancy differs based on zygosity status [12]. In addition, the FF (combined for MZ twins and individual for DZ twins) is an important determinant of the performance of NIPT for detecting aneuploidy in twin pregnancies. For MZ twin pregnancies, we observed a mean FF of 13%, which is greater than the mean FF that has previously been reported for singleton pregnancies [27]. The higher combined FF in MZ twin cases should result in the detection of aneuploidies with a performance at least as effective as that for singleton pregnancies and with a lower no-call rate. However, in DZ pregnancies, the individual FF contribution of a single fetus can be ~40% lower than observed in a singleton pregnancy [27]. Moreover, each fetus in a DZ twin pregnancy can contribute different amounts of FF (up to a two-fold difference) into the maternal circulation [28]. Thus, NIPT that only measures a combined FF is more likely to result in an incorrect call for aneuploidy risk assessment in a DZ twin pregnancy. For example, if the FF of an affected fetus is below the threshold for detection, but the total FF appears satisfactory due to a high FF contribution from the euploid co-twin, reporting based on the combined FF could lead to the incorrect assignment of a low-risk result for both fetuses. This scenario is of most concern for pregnancies where one is affected by T13 or T18 because the expected FF is significantly lower than a euploid pregnancy [29]. Given that FF is an important determinant of NIPT performance in twin pregnancies, tests that do not report individual twin FF should be interpreted with caution. Conversely, as observed in this study, methods designed to determine individual FF for DZ twins will inevitably result in no-call rates that are higher compared to singleton pregnancies, since such a method will identify those cases with individual fetal fractions too low to give a confident result [30]. Additional pregnancies where results cannot be provided include donor egg pregnancies, surrogacy, and pregnancies where there is parental consanguinity. Despite these difficulties, the data presented here add to a growing volume of studies showing overall efficacy of NIPT for aneuploidy screening in twin pregnancies is comparable to that for singleton pregnancies [22] and superior to traditional screening methods for the majority of cases where there is sufficient FF for an informative result. As for singleton pregnancies, NIPT in twin pregnancies constitutes a screening test and confirmation through invasive methods is necessary. 

## 5. Conclusions

Zygosity, fetal sex assignment, and aneuploidy assessment can be viewed as complementary evaluations in twin pregnancies that can be performed on the same specimen. The assignment of zygosity using SNPs improves estimation of aneuploidy risk because the prior risk is different based on zygosity status. The SNP-based approach is also expected to be advantageous over genome-wide counting methods for aneuploidy because of the assignment of individual fetus FFs. In addition, zygosity facilitates patient counseling regarding the likelihood that at least one of the fetuses in a twin gestation is affected. Furthermore, early assignment of zygosity can be aligned with the ultrasound assessment of chorionicity, which enables obstetric care providers to optimize individualized prenatal care management plans. 

## Supplementary Materials

The following are available online at https://www.mdpi.com/2077-0383/8/7/937/s1: Supplementary Methods 1: Study Cohort; Supplementary Methods 2: Sources of Confirmation; Supplementary Methods 3: Prior Risk Input for Aneuploidy Assessment; Supplementary Methods 4: Aneuploidy Tests—Data Analysis; Supplementary Methods 5: No-Result Rates; Supplementary Methods 6: Confidence Intervals; Supplementary Table S1: Numbers of Cases from Each of the Participating Referral Centers; Supplementary Table S2: Patient Demographics; Supplementary Figure S1: Venn Diagram of Twin Cases Included.
