# Supplementary material for: Validation of a Single-Nucleotide Polymorphism-Based Non-Invasive Prenatal Test in Twin Gestations: Determination of Zygosity, Individual Fetal Sex, and Fetal Aneuploidy
Source: J Clin Med. 2019 Jun 28;8(7):937. doi: 10.3390/jcm8070937 (PMC6679081; doi:10.3390/jcm8070937)
Supplement: Supplementary file 1 [file jcm-08-00937-s001.pdf]

Journal of
Clinical Medicine

MDPI

Supplementary Information

Methods 1. Study Cohort

Pregnant women were enrolled at 21 locations or centers: Columbia University, New York, NY; Houston Perinatal Associates, Houston, TX; San Diego Perinatal Center, San Diego, CA; San Gabriel Valley Medical Center, San Gabriel, CA; Carnegie Hill Imaging for Women, New York, NY; Texas Children's Hospital, Houston, TX; Lyndhurst Gynecologic Associates, Winston-Salem, NC; The Woman's Hospital of Texas, Houston, TX; Saint Peter's University Hospital, New Brunswick, NJ; Tufts Medical Center, Boston, MA; Montefiore Medical Center, Bronx, NY; Mount Sinai Health System, New York, NY; Center for Maternal-Fetal Health, New Hyde Park, NY; Northwestern University, Evanston, IL; StemExpress, Placerville, CA; Natera Inc., San Carlos, CA; Ospedale Maggiore Policlinico, Milano, Italy; Ospedale Michele Sarcone, Bari, Italy; Ospedale Piero Palagi, Firenze, Italy; Hospital Vall d'Hebron, Barcelona, Spain; International Kent Hospital, Izmer, Turkey between April 2013 and February, 2017. A sample of maternal blood (approximately 20 mL) was obtained at ≥9 weeks' gestational age (GA). All sample collection was conducted in accordance with local laws and institutional review board-approved protocols from each participating site and all women provided written informed consent. Maternal age and GA were collected at the time of blood draw. Samples and data were collected and stored by a unique study identification code number to protect participant confidentiality.

Inclusion criteria included over 18 years of age at time of enrollment, sonographically confirmed twin pregnancy, gestational age (GA) between 9–26 weeks by best obstetrical estimate, and willingness to provide signed informed consent. A subset of the cohort (n = 56) was enriched for aneuploidy cases and satisfied one of the following additional inclusion criteria: Confirmed affected with aneuploidy by invasive testing, non-invasive prenatal test (NIPT) "high-risk" result, serum screening risk of greater than 1:100, or observed ultrasound abnormalities suggestive of aneuploidy. Exclusion criteria included women with singleton pregnancies or the use of a surrogate or egg donor.

Methods 2: Sources of "Truth"

The true presence or absence ("truth") of MZ, DZ, aneuploidy (T21, T18, T13, monosomy X), or 22q11.2 deletion and the fetal sex were based on the following information: For the 95 cases tested for zygosity, MZ versus DZ was determined by molecular genetic testing by an external laboratory (n = 47), presence of twins with different fetal sex (n = 36, only valid for DZ), SNP-based analysis of buccal samples from children (n = 8), clinical presentation of twin-to-twin transfusion syndrome (TTTS) (n = 3), or single embryo transfer plus monochorionic/monoamniotic observation by ultrasound (n = 1).

For the 117 cases tested for aneuploidy, truth was determined by cytogenetic or molecular genetic testing by an external laboratory (n = 50), SNP-based analysis of buccal samples from children (n = 28), visual inspection (not applicable to monosomy X) (n = 36) or a combination of the aforementioned methods (n = 3). For the 103 cases tested for fetal sex, truth was determined by visual inspection of neonates (n = 46), cytogenetic or molecular genetic testing by external laboratory (n = 33), SNP-based analysis of buccal samples from children (n =22), or a combination of the aforementioned methods (n = 2). These criteria were independently recorded and verified by a centralized team of site monitors.

Methods 3. Prior Risk Input for Aneuploidy Assessment

Prior risk of aneuploidy for twin pregnancies was first assigned from the singleton prior risk, which is based on maternal age and gestational age. For each twin pregnancy, the prior risk was then adjusted based on the assigned zygosity. The prior risk for MZ pregnancies is the singleton risk

adjusted by a factor of 0.336; for DZ pregnancies the prior risk is the singleton risk adjusted by a factor of 1.5.[1]

Methods 4. Aneuploidy Tests—Data Analysis

DZ aneuploidy sensitivity was defined as the proportion of confirmed affected DZ samples with a call that was positive for T21, T18, or T13 by the algorithm. MZ aneuploidy sensitivity was not determined due to insufficient sample numbers (n = 1). Aneuploidy specificity for DZ samples was defined as the proportion of confirmed unaffected DZ samples with a call that was negative for T21, T18, and T13 and for MZ samples as the proportion of confirmed unaffected MZ samples with a call that was negative for T21, T18, T13, and MX. Overall aneuploidy specificity was estimated using an MZ:DZ weighted average.

Methods 5. No-Result Rates

No-result rates were defined as the proportion of samples with truth that did not receive a result, and were estimated using a MZ:DZ weighted average. No-result rates for the zygosity and fetal sex tests included all tested samples with GA ≥9 weeks; no-result rates for the aneuploidy tests included all tested euploid samples with GA ≥10 weeks for ease of comparison to other clinically available NIPT methods.

Methods 6. Confidence Intervals

For the overall aneuploidy specificity estimate, fetal sex accuracy, aneuploidies test no-result rate, and fetal sex test no-result rate calculations, confidence intervals (CI) of the true weighted average were computed using the Method of Variance Estimates Recovery (MOVER) based on exact binomial Clopper–Pearson confidence intervals for a single proportion. Confidence intervals for all other test performance estimates are exact binomial Clopper–Pearson confidence intervals for a single proportion.

Table S1. Number of cases tested for zygosity, fetal sex, and aneuploidy from each of the 21 locations.

Site	Zygosity	Sex	Aneuploidy
1	10	11	12
2	1	1	1
3	1	0	1
4	1	2	2
5	0	2	2
6	25	30	33
7	1	1	1
8	1	0	1
9	1	1	1
10	3	3	3
11	1	1	1
12	2	2	3
13	4	3	4
14	23	22	23
15	0	4	4
16	1	1	1
17	5	3	5
18	3	3	3
19	1	1	1
20	11	11	11
21	0	1	4
**Total**	**95**	**103**	**117**

Table S2. Patient demographics.

	Zygosity	Fetal Sex	Aneuploidy
Total cases	95	103	117
MZ:DZ	30:65	40:63	40:77
Maternal age (mean ± SD)	32.8 ± 5.2	32.8 ± 5.3	33.0 ± 5.5
Gestational age (mean ± SD)	15.4 ± 4.7	15.4 ± 4.6	15.6 ± 4.8

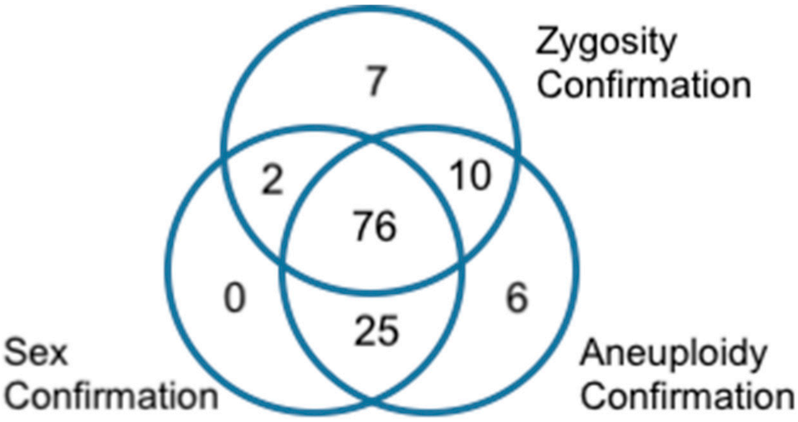

Figure S1. Venn diagram to show the overlapping groups of cases used in the study.

References

1. Sparks TN, Norton ME, Flessel M, Goldman S, Currier RJ. Observed Rate of Down Syndrome in Twin Pregnancies. Obstetrics and Gynecology. 2016;128(5):1127-33.
